# Prognostic factors of traumatic optic neuropathy based on multimodal analysis—Especially the influence of postoperative dressing change and optic nerve blood supply on prognosis

**DOI:** 10.3389/fneur.2023.1114384

**Published:** 2023-01-30

**Authors:** Xueru Liu, Jing Wang, Wenyue Zhang, Lunhao Li, Leilei Zhang, Caiwen Xiao

**Affiliations:** ^1^Department of Ophthalmology, Shanghai Ninth People's Hospital, Shanghai Jiao Tong University, School of Medicine, Shanghai, China; ^2^Shanghai Key Laboratory of Orbital Diseases and Ocular Oncology, Shanghai, China

**Keywords:** continuous treatment, multimodal analysis, optic nerve blood supply, predictive model, ETOCD

## Abstract

**Objective:**

To investigate the critical prognostic factors of patients with traumatic optic neuropathy (TON) treated with endoscopic transnasal optic canal decompression (ETOCD) and to perform multimodal analysis based on imaging examinations of optical coherence tomography angiography (OCTA) and CT scan. Subsequently, a new prediction model was established.

**Methods:**

The clinical data of 76 patients with TON who underwent decompression surgery with the endoscope-navigation system in the Department of Ophthalmology, Shanghai Ninth People's Hospital from January 2018 to December 2021 were retrospectively analyzed. The clinical data included demographic characteristics, reasons for injury, interval between injury and surgery, multimode imaging information of CT scan and OCTA, including orbital fracture, optical canal fractures, vessel density of optic disc and macula, and the times of postoperative dressing change. Binary logistic regression was used to establish a model for best corrected visual acuity (BCVA) after treatment as a predictor of TON outcome.

**Results:**

Postoperative BCVA improved in 60.5% (46/76) patients and did not improve in 39.5% (30/76) patients. The times of postoperative dressing change had a significant impact on the prognosis. Other factors affecting the prognosis were microvessel density of the central optic disc, the cause of injury, and the microvessel density above the macula. The area under the raw current curves of the predictive model was 0.7596.

**Conclusions:**

The times of dressing changes after the operation, i.e., continuous treatment, is the key factor affecting prognosis. The microvessel density in the center of the optic disc and superior macula, quantitatively analyzed by OCTA, is the prognostic factor of TON and may be used as a prognostic marker of TON.

## Introduction

TON refers to the optic nerve injury caused by external violence during cranial or facial trauma. The clinical characteristics of TON are acute or progressive visual loss and/or visual field defect after trauma (1). The mean age of the patients was 31 years, and 78.5% were males. The primary causes were fall injury, road traffic accident, and fight (2, 3).

Optic canal decompression surgery and glucocorticoid pulse therapy are the primary methods for TON treatment (4, 5). However, due to the lack of a large sample and multicenter randomized controlled trial results, there is still no standard treatment strategy for the treatment of TON in recent years.

Our previous retrospective study showed positive outcomes with ETOCD for patients with TON, especially those with good BCVA before treatment, short intervals between trauma and treatment, no orbital fracture or ethmoid, and/or sphenoid sinus bleeding (6). In addition, a visual nomogram prediction model was established by binary logistic regression, but in clinical practice, this model might not include all relevant factors affecting prognosis. Are there other key factors? We found that the blood supply of the optic nerve and the continuous treatment after surgery may have some influence on the prognosis.

In this study, OCTA was used to quantitatively detect peripapillary capillary and macular capillary density to directly reflect the blood supply of the optic nerve. Then, the condition and damage degree of patients were analyzed with traumatic optic neuropathy based on OCTA and CT polymorphic imaging (7, 8).

In addition to surgical factors, the prognosis of patients with TON may also be closely related to long-term postoperative treatment and the application of nerve growth factors. In the standard treatment process of this study, patients were treated with postoperative dressing change, and this retrospective study determined whether it had an effect on the prognosis (9).

In summary, the current study analyzed the prognostic factors of TON patients treated with ETOCD based on OCTA and CT multimodal images through new detection indicators and standard treatment procedures and identified the key factors affecting the prognosis. These laid the foundation of a new prediction model.

## Methods

### Study design and patients

This retrospective cohort study comprised of samples 76 patients who underwent ETOCD with ENS in the Department of Ophthalmology, Ninth People's Hospital, Shanghai Jiao Tong University School of Medicine, Shanghai, China from January 2018 to December 2021.

Surgical indications/inclusion criteria: History of trauma, traumatic brain injury or active bleeding without affecting vital signs, visual loss after injury (immediately or delayed after injury), optic canal fracture or submucosal sphenoid hemorrhage on computed tomography (CT) scan, relative afferent pupillary defects (positive).

Exclusion criteria: Patients with previous optic nerve injury or other ophthalmic diseases that may affect vision; severe traumatic brain injury affecting vital signs; follow-up was <1 month; data for the primary outcome (visual acuity) were missing.

The patients were independently diagnosed by two senior ophthalmologists (CX and XF). All ETOCD procedures were conducted by the same ophthalmologist (CX). Gender, age, reason of injury, orbital fracture, optic canal fracture, ethmoid and/or sphenoid sinus bleeding on CT, the interval between trauma and treatment, and inner retinal capillary density on OCTA were recorded. The data were divided into the middle, upper, lower, temporal, and nasal regions, BCVA before and after treatment, the times of postoperative dressing change (To avoid bias due to both the doctor's decision and the patient's demand, we typically required three dressing changes for each patient).

### Treatment

#### Before surgery

Methylprednisolone (30 mg/kg/day) was administered for 3 days before treatment, and mouse-derived nerve growth factor (NGF) (30 μg/mL/ day) was used daily before surgery (Staidson, Beijing, China).

#### Surgical procedures

**Figure 1:**
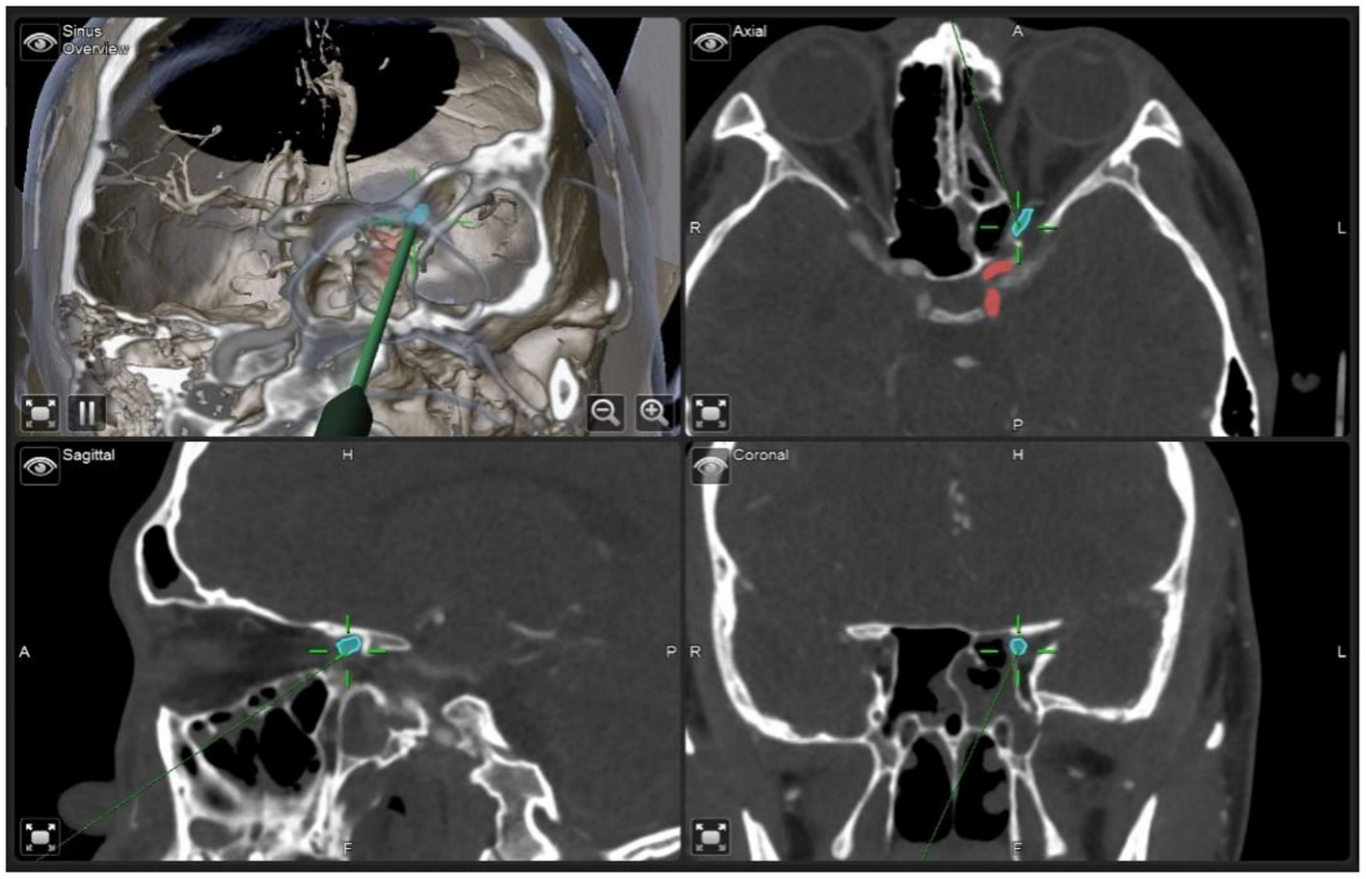
Real-time positioning of the optic nerve and carotid artery accurately displayed by navigation during ENS. Blue: The optic nerve; Red: Internal carotid artery.

ENS plan was designed according to preoperative carotid artery angiography data. The relative position of the optic nerve of the internal carotid artery was marked clearly. Under general anesthesia, a transnasal endoscopic sphenoid ethmoidectomy was performed. The uncinate process and ethmoid bulb were removed to expose the posterior ethmoid sinus. Subsequently, superior turbinectomy was performed to expose the anterior wall of the sphenoid sinus for further surgery. Under the guidance of ENS plan, the position and direction of the optic nerve, the cranial opening of the optic nerve, and the medial wall of the internal carotid artery of the optic canal were well-positioned (Figure 1). Then, the 180° medial superior and medial inferior walls of the optic canal were thinned using a microdrill, and the optic canal and nearby orbital apex bone were dissected with a stripping device to fully expose the underlying optic nerve. The optic nerve sheath was cut with a bayonet, the incision was filled with nasopore, and 30 μg NGF and 1 mL triamcinolone acetonide were injected.

#### Postoperative treatment

Methylprednisolone (30 mg/kg/day) was used immediately after the operation and then decreased to 15 mg/kg/day the next day; Cefadroxil (4.0 g/day) for 3 days; NGF 30 μg/day intramuscular injection for 14 days as a course of treatment.

#### Dressing change

**Figure 2:**
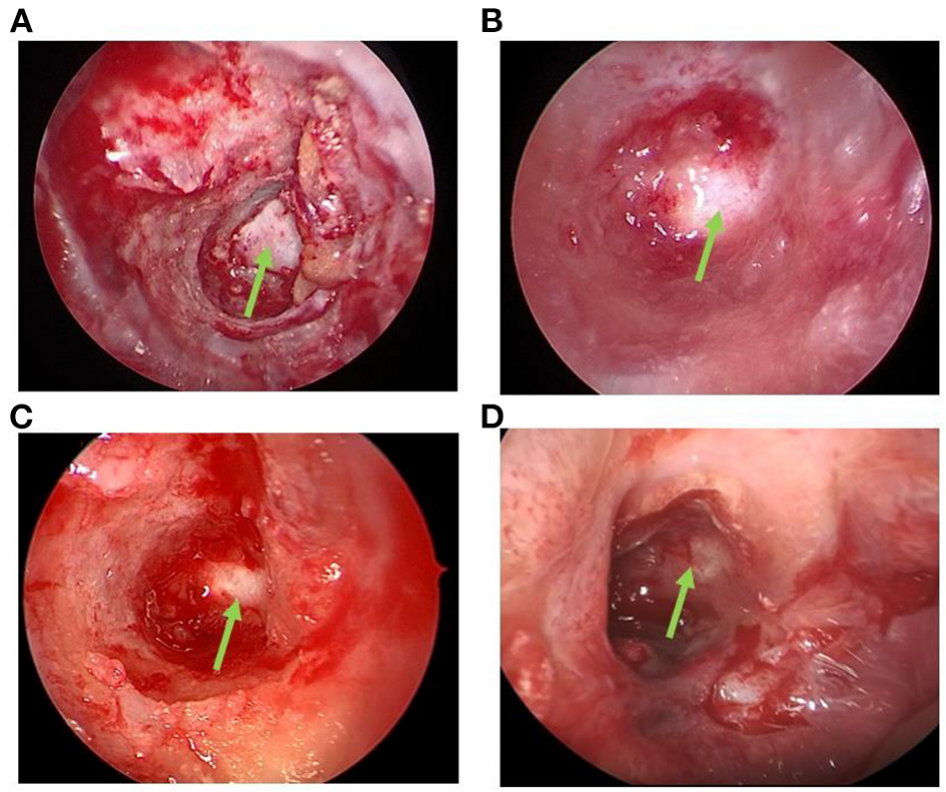
Green arrow: The optic nerve. **(A)** Immediately after decompression: The optic nerve was exposed, and the decompression was sufficient. **(B)** After dressing change 1 week after surgery: Sphenoid sinus and ethmoid sinus were cleaned, secretion was removed by suction, nasopore was filled beside the optic nerve, and 30 μg NGF+1 mL triamcinolone acetonide was injected. **(C)** After dressing change 3 weeks post-surgery. **(D)** After the dressing change 5 week post-surgery, there was no secretion in the sinus cavity, and the mucosa was healed.

patients were required to complete 3 times of dressing change after operation, respectively at 1, 3, and 5 weeks after operation. The brief process of dressing change was as follows: cleaning the surgical cavity, including sphenoid sinus and ethmoid sinus, and removing the secretions in the surgical cavity under nasal endoscope. The most critical step is filling near the optic nerve with nasopore, and 30 NGF and 1 mL triamcinolone acetonide were injected (Figure 2).

### Visual acuity evaluation index

All patients included in this study underwent preoperative and postoperative visual acuity examination based on a standard logarithmic visual acuity chart and comprehensive refractometer and obtained the best corrected visual acuity (BCVA; standard logarithmic visual acuity chart).

In this study, visual acuity was divided into five levels: 1. No light perception (NLP), 2. Light perception (LP), 3. Hand motion (HM), 4. Finger counting (FC), 5. Standard logarithmic visual acuity chart.

Visual improvement: The postoperative visual acuity was improved by one level compared to that before surgery, For patients with preoperative visual acuity of level 5, postoperative visual acuity was improved by two lines.

### Statistical methods

Continuous variables are described as means ± standard deviations or medians (interquartile range, IQR), depending on data distribution. *T*-test or non-parametric rank-sum test was used for comparison between groups. Categorical variables were described as frequencies (percentages). Chi-square test was used to compare the rates between groups.

In predictive model training, dummy variables were set in BCVA. Binary logistic regression was used to analyze the influencing factors of postoperative BCVA. The results of logistic regression analysis were graphically displayed using a nomogram, and the diagnostic and predictive value of the nomogram was evaluated using receiver operating characteristic (ROC) curves.

## Results

**Figure 3:**
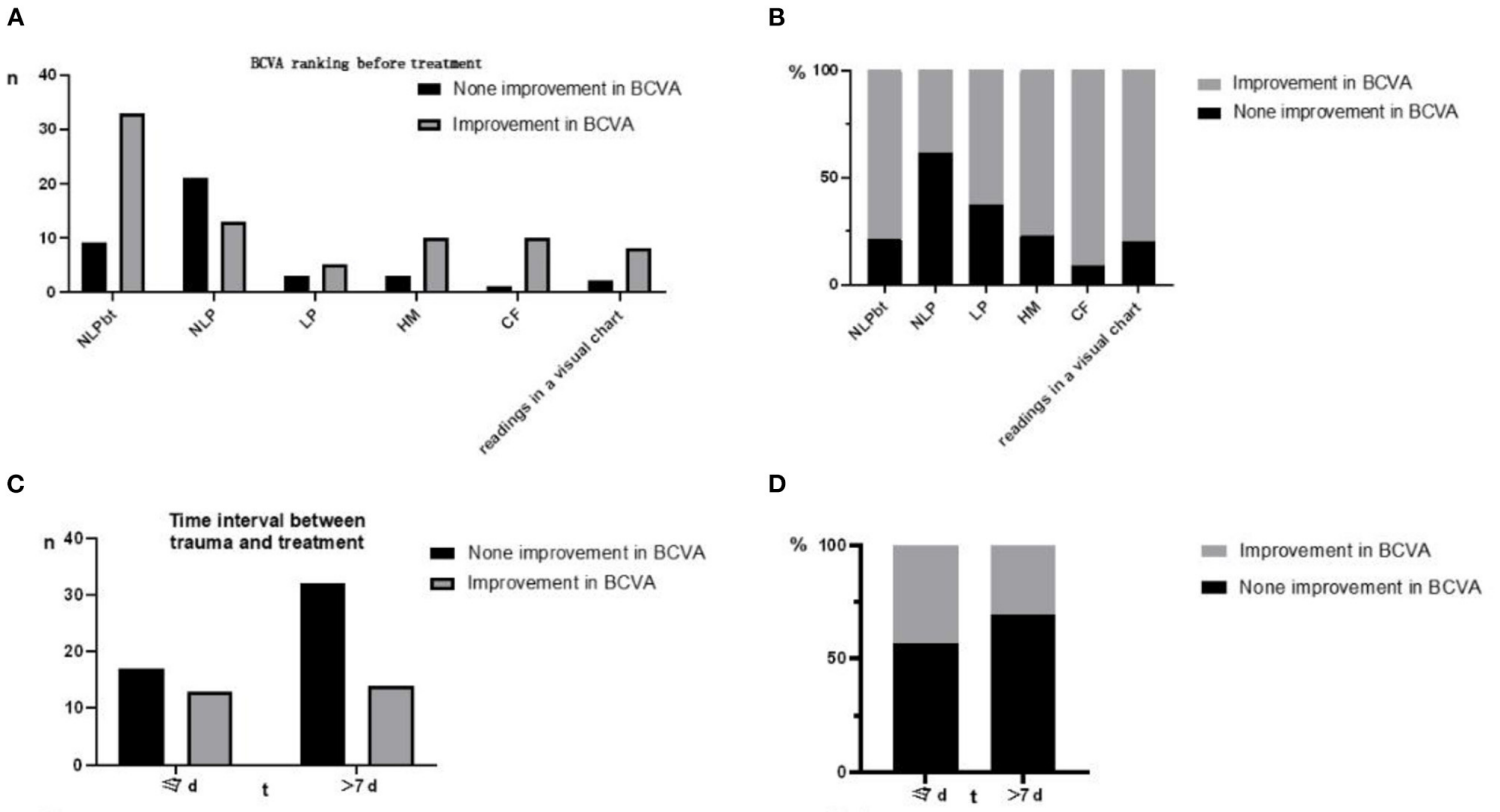
**(A)** Distribution of preoperative visual acuity in TON patients. **(B)** The higher the preoperative visual acuity level, the higher the postoperative improvement rate. 78.6% of btNLP patients improved, which was higher than 38.5% of NLP patients. The difference between the two groups was statistically significant (*P* < 0.001). **(C)** Interval between trauma and surgery in TON patient. **(D)** 66% of patients who underwent surgery within 7 days showed improvement, and 51.8% of patients who underwent surgery beyond 7 days showed improvement. No statistical difference was observed between the two groups.

**Figure 4:**
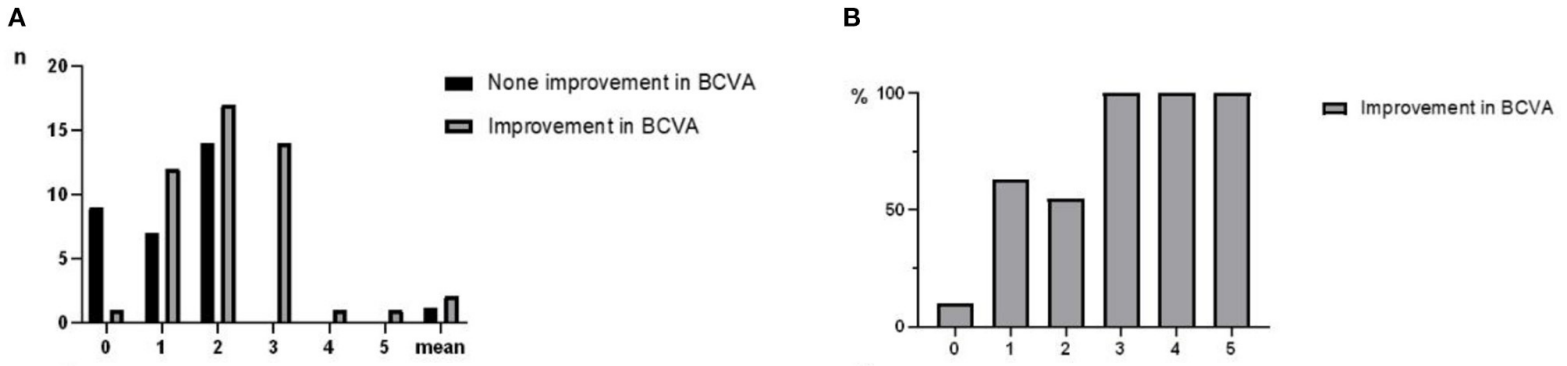
**(A)** Times of dressing change in TON patients after surgery: The average time of dressing change in the improved group was 2.11 times, and that in the non-improved group was 1.17 times. **(B)** Times of dressing change were significantly correlated with prognosis (***P < 0.001).

**Figure 5:**
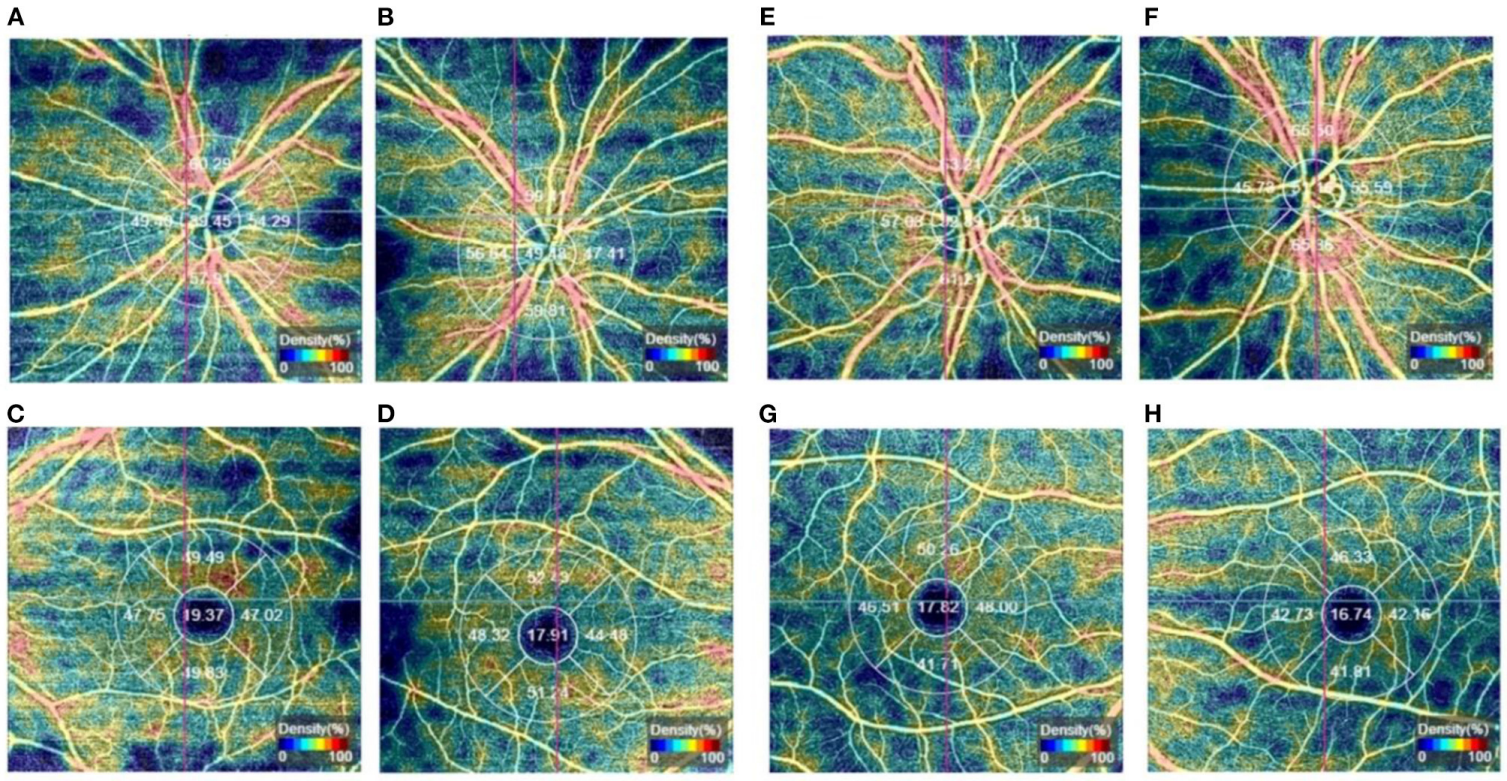
**(A–D)** One patient in the non-improvement group, **(A, C)** are the affected eyes, the microvessel density in the center of the optic disc and superior macula was significantly lower than that of the healthy eye; **(B, D)** are the healthy eyes; **(E–H)** One patient in the improvement group, **(E, G)** are the affected eyes, **(F, H)** are the healthy eyes.

Between January 2018 and December 2021, 76 subjects were screened for eligibility and followed up for 6.4 (6–30) months. 46/76 (60.5%) patients showed an improvement in BCVA after treatment, while 30/76 (39.5%) had no improvement. 33/42(78.6%) of patients with better preoperative visual acuity than NLP (btNLP) had improved BCVA after surgery, while 13/34 (38.2%) of patients with NLP had improved BCVA after surgery (Figure 3); a significant difference was detected between the two groups. The patients who showed improvement after surgery had an average of 2.11 times dressing change (0–5 times), and the patients who did not improve had an average of 1.17 times dressing change (0–3 times) (Figure 4). Patients with more dressing changes after surgery had a high improvement rate. The preoperative blood flow density of the optic disc and macula is a factor affecting the prognosis. Patients with a low decrease in blood flow density, i.e., relatively better blood supply of the optic nerve, are likely to recover after surgery (Figure 5, data of all patients not shown). The cause of injury is one of the factors affecting the prognosis. Patients injured by heavy objects in traffic accidents are likely to recover. The demographic characteristics and preoperative statistics of the patients are summarized in Table 1.

**Table 1:** Clinical features of the patients.

	**Category**	**No improvement in BCVA**	**Improvement in BCVA**	***P***
*n*		30 (39.5)	46 (60.5)	
Gender (%)	M	25 (32.9)	41 (53.9)	0.503
	F	5 (6.6)	5 (6.6)	
Age (mean)		35.32	34.83	0.882
Cause	Fall down	6	7	0.55
	Motorcycle accidents	10	21	
	Car accident	5	10	
	Fall from height	3	5	
	Hurt by a heavy object	3	1	
	Others	3	2	
Orbital fracture (%)	+	27 (35.5)	39 (51.3)	0.731
	–	3 (3.9)	7 (9.2)	
Optic canal fracture (%)	+	9 (11.8)	15 (19.7)	0.811
	–	21 (27.6)	31 (40.8)	
Interval between trauma and treatment (%)	≤7	17 (34.7)	32 (48.1)	0.152
	>7	13 (65.3)	14 (51.9)	
Hemorrhage within the post-ethmoid and/or sphenoid sinus (%)	+	13	13	0.134
	–	17	33	
BCVA ranking before treatment (%)	NLP	21	13	0.005
	LP	3	5	
	HM	3	10	
	CF	1	10	
	Readings in a visual chart	2	8	
BCVA ranking after treatment (%)	NLP	25	0	<0.005
	LP	2	4	
	HM	1	3	
	CF	0	7	
	Readings in a visual chart	2	32	
Times of dressing change	0	9	1	<0.005
	1	7	12	
	2	14	17	
	3	0	14	
	4	0	1	
	5	0	1	

**Figure 6:**
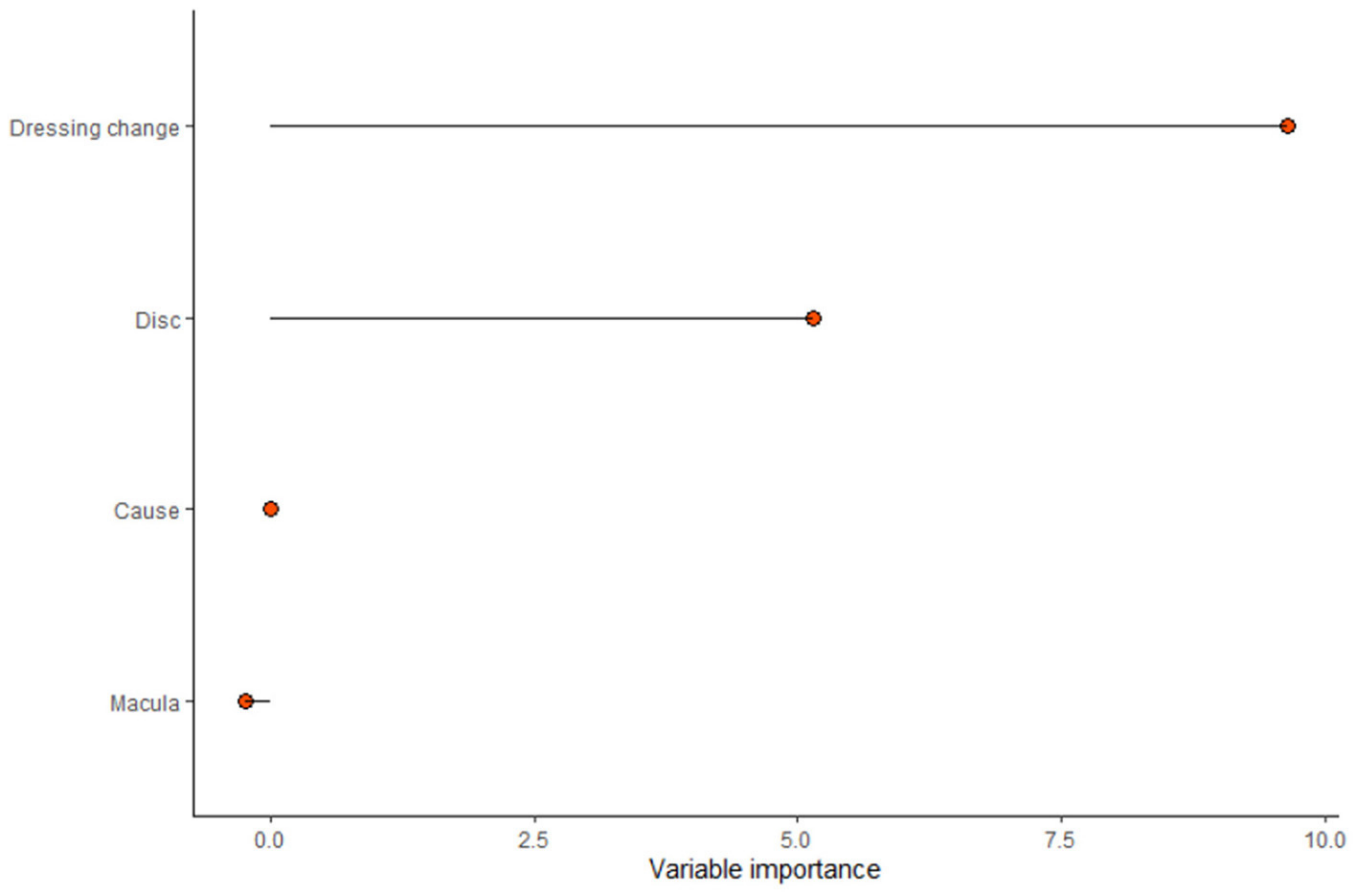
The importance of factors affecting prognosis: The most important was the times of dressing change, followed by the central microvessel density of the optic disc, the cause of injury, and the microvessel density above the macula.

**Figure 7:**
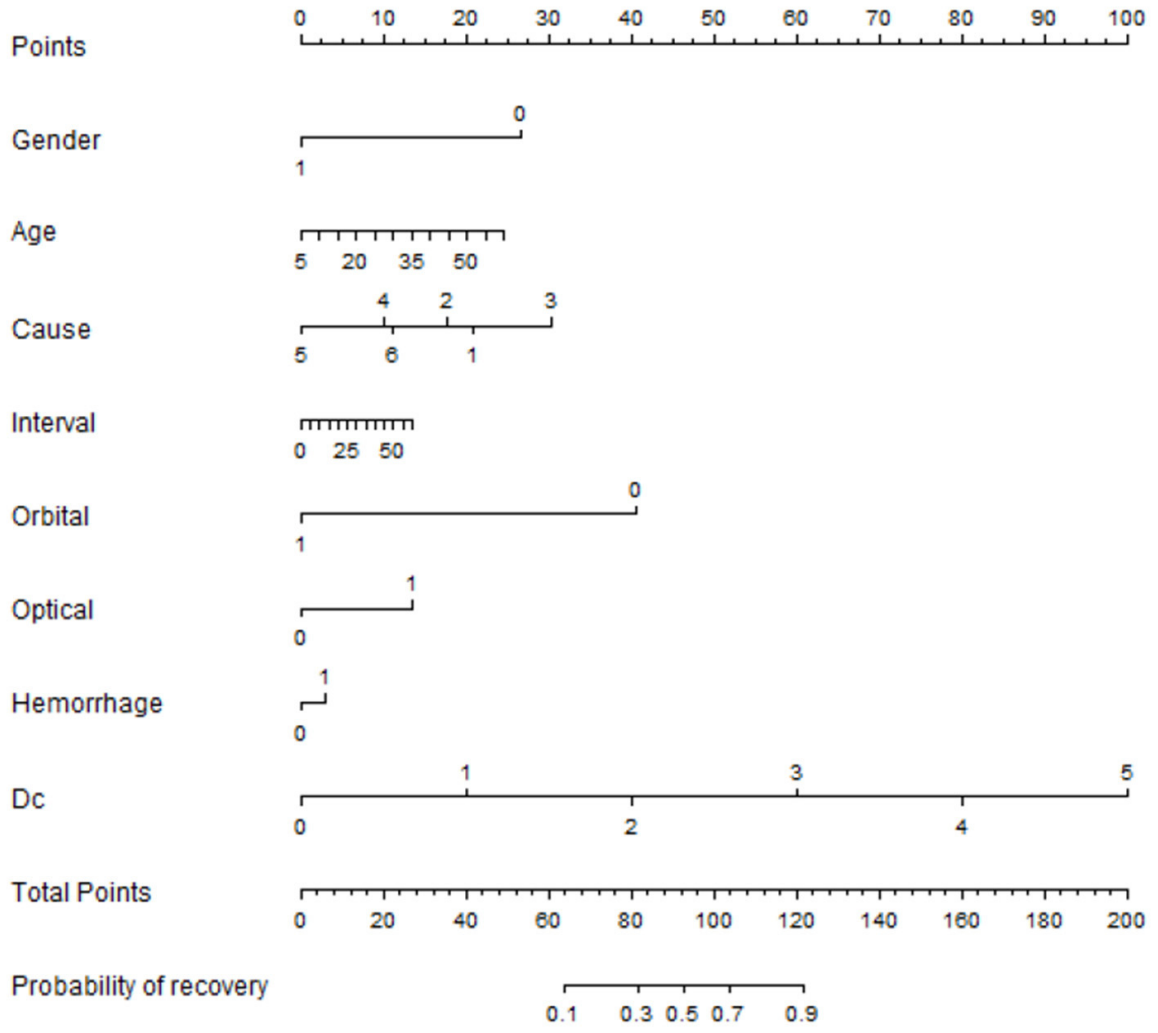
Prediction model nomogram: A score of 95 predicted that the probability of postoperative improvement was 50%. Dc: times of dressing change.

In this model, the times of dressing change, microvessel density in the center of the optic disc, cause of injury, and microvessel density above the macula were include as predictors (Figure 6). For this model, the area under the curve (AUC) = 0.7596, specificity = 0.6250, and sensitivity = 0.7692. The nomogram of this model is shown in Figure 7.

## Discussion

The purpose of surgical treatment of TON is to relieve the mechanical compression of the visual pathway, improve local blood circulation, and prevent further damage (10). It is crucial for clinicians to evaluate the condition, choose the appropriate treatment before the operation, and judge the prognosis to clarify the critical factors affecting the prognosis of TON patients (11, 12). However, secondary injury and repair and regeneration after optic nerve injury is a long-term and chronic process. Previous studies have focused on preoperative visual acuity and CT examination and rarely focused on the effect of the optic nerve blood supply and continuous postoperative medication on prognosis (13, 14). This is the first study to focus on the effect of blood supply and postoperative treatment on prognosis in addition to preoperative evaluation.

Dressing change with NGF and triamcinolone acetonide is a critical prognostic factor in this study (Figure 6), and patients with several courses had high scores for improved outcomes. This finding could be attributed to two possible reasons. First, patients with improved visual acuity have higher compliance and adhere to a longer course of treatment than patients without improved visual acuity. Second, patients with no improvement had more severe injuries and a greater proportion of NLP than those with improvement, no improvement in visual acuity after surgery, and no obvious drug effect. On the other hand, significant differences were observed between the two groups, which proved that continuous postoperative treatment plays a crucial role in prognosis. Dressing change, a patch of nasopore next to the optic nerve, and injection of NGF and triamcinolone acetonide had a positive effect on the prognosis. The mechanism of action is to improve the microenvironment of nerve injury and promote nerve recovery (15). Due to the obstruction of epineurium and blood-nerve barrier, the concentration of drugs reaching the nerve injury is not high (16), such that nanopore-mediated degradable materials are attached to the optic nerve to increase the concentration of drugs and promote nerve recovery. NGF increases the activity of nerve growth factor in the injured nerve by acting on the high-affinity receptor tyrosine kinase and low-affinity receptor of effector cells regulates the microenvironment for the survival of neurons, protects the survival of neurons, and promotes myelin repair (17, 18). Triamcinolone acetonide prevents adhesion and scar formation by reducing exudation and edema in the early stage of inflammation, inhibiting free radical damage, and reducing capillary permeability in the later stage of inflammation (19, 20).

Ma et al. applied a gelatin sponge infiltrated with dexamethasone and NGF to optic nerve injury and found that it promoted optic nerve repair (21). These results were consistent with our results, suggesting that in clinical practice, the completion of surgery does not indicate the end of treatment for TON patients, and we should actively adhere to continuous medication after surgery to obtain a better prognosis. Since the optic nerve fiber can regenerate in a specific period when the optic nerve fiber is not completely damaged, it is necessary to relieve the compression of the optic canal by surgery in the early stage, followed by long-term use of drugs to promote nerve repair and regeneration to improve the prognosis of patients.

Another prognostic factor identified in this study was the degree of reduction in the central microvessel density of the optic disc. Optic nerve injury is caused by externally transmitted mechanical shear stress after trauma (22). Shear stress can destroy the axons of some retinal ganglion cells and the peripheral blood vessels supplying the optic nerve. The OCTA detection of retinal microvessel density is a new non-invasive vascular imaging method that uses the algorithm of frequency division enhanced coherence angiography (23–25).

In this study, we performed bilateral OCTA in patients with TON and found that the blood flow signal in and around the optic disc of the affected eye was significantly lower than that of the healthy eye immediately after injury, and the microvessel density was also significantly lower than that of the healthy eye. Simultaneously, the blood flow signal in the macular area of the patient's affected eye was also reduced to varying degrees compared to the healthy eye (Figure 5). These findings were pronounced in patients with severe optic nerve damage, low vision, or even blindness. In future clinical research, we would include several samples compared to normal people and use microvessel density as a prognostic marker for TON patients.

In this study, microvessel density in the center of the optic disc and in the superior macula was an independent prognostic factor (Figure 6). However, in this study sample, 14 patients lacked the data, and the prediction result was poor even after including the data in the prediction model. If this subset of the sample data is added to the prediction model, the ROC value of the prediction model may be improved.

The present study, for the first time, showed that the cause of injury may be associated with outcomes. Worldwide epidemiological studies have shown that the main causes of TON are falls, road traffic accidents, and fights (2, 3). In addition, patients injured by heavy objects in traffic accidents had improved prognosis and high preoperative scores (Figure 7). This phenomenon could be attributed to cranial and facial trauma occurs, especially in the eyebrow arch and frontal and temporal trauma; external impact force will be transmitted to the optic nerve through the bony structure of the focus, resulting in contusion necrosis of the optic nerve. The main injury in traffic accidents is blunt craniocerebral trauma, while heavy object injury and falling injury caused by electric bicycles mainly focus on the frontotemporal region. The impact force transmitted to the optic canal is large, especially for patients with serious heavy object injury to the frontotemporal region.

Previous studies have shown that preoperative visual acuity and the interval between injury and surgery are independent factors associated with prognosis (26, 27). The current results showed that patients with better preoperative visual acuity than those with no light perception, shorter duration of injury, and surgery have a higher rate of high postoperative visual acuity (Figure 3). Although in this dataset, the difference between groups may be due to the small sample size and the difference between groups. It is not an independent factor but suggests that for patients with NLP, the indications for surgery should be strictly controlled, and conservative treatment should be adopted for patients with little possibility of improved prognosis. For patients who cannot be treated in time due to other reasons, ETOCD treatment can still be used to try to save the impaired vision if the patient has a strong will because there are still some patients whose operation time has been >7 days when they visit the hospital, but the postoperative vision is improved.

The BCVA results of our surgical intervention with ENS in TON patients were satisfactory. Postoperative visual acuity was improved in 60.5% of patients, and the improvement rate was high in btNLP patients (78.6%). CT combined with OCTA multimode imaging analysis was used to evaluate the patient's condition, and all the relevant factors that may affect the prognosis were analyzed to obtain the factors with a significant effect on the prognosis. In the treatment process of patients, the importance of postoperative treatment for the prognosis was emphasized. Based on new research data, a prediction model with AUC = 0.7596 was established, which could be used as a judgment tool (Figure 7). Patients with scores >95 are expected to improve BCVA after ENS, while in those with scores <95, BCVA improvement rate is expected to be <50%; thus, we favor conservative management.

## Conclusions

Postoperative continuous medication is crucial for the prognosis of TON patients. After a comprehensive evaluation of the condition combined with CT, OCTA multimodal imaging information, and clinical manifestations of patients, the patients with a higher recovery rate should be selected for surgery, and continuous treatment should be administered after surgery. The limitations of this study are the small sample size and the lack of a randomized controlled trial (RCT). Interestingly, future studies would focus on the NGF treatment and blood flow changes, including a large number of samples, blood flow changes, and incorporating OCTA results into the prediction model.

## Data availability statement

The original contributions presented in the study are included in the article/supplementary material, further inquiries can be directed to the corresponding author.

## Ethics statement

The studies involving human participants were reviewed and approved by the study protocol was approved by the Ethics Committee of Shanghai Ninth People's Hospital (No. SH9H-2018-T31-1) in accordance with the Declaration of Helsinki 2015. Written informed consent to participate in this study was provided by the participants' legal guardian/next of kin.

## Author contributions

Conceived and designed the study: CX. Acquisition of data: XL, JW, and WZ. Analysis and interpretation of data: XL, LZ, and LL. Drafting the manuscript: XL and JW. All authors have read and approved the final manuscript.
